# Radiological assessment of chest compression point and achievable compression depth in cardiac patients

**DOI:** 10.1186/s13049-016-0245-0

**Published:** 2016-04-22

**Authors:** Sverre Nestaas, Knut Haakon Stensæth, Vigdis Rosseland, Jo Kramer-Johansen

**Affiliations:** Department of Anaesthesiology, Oslo University Hospital, Po Box 4956, Nydalen, N-0424 Oslo, Norway; Department of Radiology and Nuclear Medicine and Institute of Circulation and Imaging, St Olavs University Hospital and Norwegian University of Science and Technology, Po Box 3250, Sluppen, N-7006 Trondheim, Norway; Intervention- and Sonography Unit, Oslo University Hospital, Po Box 4956, Nydalen, N-0424 Oslo, Norway; Norwegian National Advisory Unit on Prehospital Emergency Medicine (NAKOS), Oslo University Hospital and University of Oslo, Po Box 4956, Nydalen, N-0424 Oslo, Norway

**Keywords:** Cardiopulmonary resuscitation, Chest compression, Hand position, Anatomical landmark, Compression depth, Magnetic resonance imaging (MRI)

## Abstract

**Background:**

Using magnetic resonance imaging (MRI) to relate cardiovascular structures to surface anatomy in a population relevant to cardiac arrest victims, relate the external thoracic anterior-posterior (AP) diameter (AP_EXTERNAL_) and blood-filled structures to recommended chest compression depths, and define an optimal compression point (OCP).

**Methods:**

MRI axial scans of referred patients were analysed. We defined origo as the skin surface of the centre of sternum in the internipple line. The blood-filled structures beneath origo were identified and the sum of their inner diameters (AP_BLOOD_) and AP_EXTERNAL_ were measured. We defined OCP based on the image with maximum compressible left and right ventricle and where LVOT was not present. We measured the distance from origo to OCP.

**Results:**

Consecutive patients, mean (SD), age 52 (17) years, 110 (76 %) males, were categorized: cardiac disease (*n* = 74), aortic disease (*n* = 13), no findings/study patient (included in another study) (*n* = 57). The structure LVOT/aortic valve (AV)/aortic root was present in 46 % of patients with cardiac disease vs. 19 % of patients with no findings. AP_EXTERNAL_ for males and females was 25 (2) cm and 22 (2) cm, and AP_BLOOD_ 6.5 cm (2) and 4.7 cm (2), respectively. Distance from origo to OCP was 32 (11) mm to the left and 16 (21) mm caudally.

**Discussion:**

LVOT/AV/aortic root was present beneath the origo in almost half the patients with cardiac disease. Recommended chest compression depths exceeded the anterior-posterior diameter of blood-filled structures in more than half of the females. OCP was found 3 cm left of the origo.

**Conclusions:**

Based on our study, individualized compression point and depth could be further studied in a prospective, clinical study.

**Electronic supplementary material:**

The online version of this article (doi:10.1186/s13049-016-0245-0) contains supplementary material, which is available to authorized users.

## Background

Effectiveness of chest compressions during cardiopulmonary resuscitation (CPR) can be altered by hand position and compression depth, among other components [1]. The International Liaison Committee on Resuscitation (ILCOR) recommends placing the hands on the lower half of the sternum and concluded in 2010 that the use of the internipple line (INL) as a landmark for hand placement is not reliable [1, 2]. However, there is little scientific evidence regarding the optimum hand position for chest compressions and the INL is an easily detectible surface landmark. Altered hand position might change compression of intrathoracic structures. Forward blood flow during CPR is provided by compression of the heart (cardiac pump theory) and/or intra-thoracic pressure fluctuations (thoracic pump theory). According to the cardiac pump theory, direct compression of the ventricles is important. Previous CT studies have however shown that rather than the left ventricle, structures beneath the INL have been the ascending aorta, the root of aorta, or the left ventricular outflow tract (LVOT) [3]. Compression of the base of the heart or the LVOT might impede forward blood flow [4, 5], whereas compression of both ventricles avoiding LVOT might be more effective. As to compression depths, European Resuscitation Council (ERC) decided in 2015 to retain the 2010 guidance that chest compressions should be at least 5 cm but not more than 6 cm [6], which equates to approximately one-fifth of the adult chest [7]. The ERC recommendations are regardless of gender, body size, and medical history.

The purpose of this study was to establish how cardiovascular structures known to affect CPR, relate to surface anatomy in patients referred to cardiac magnetic resonance imaging (MRI). We then related the external anterior-posterior (AP) diameter of both the thorax and of its blood-filled structures, to the recommended chest compression depths. Finally, we defined a hypothesis generating optimal compression point (OCP) based on maximum compressible left and right ventricle.

## Methods

### Setting and participants

This was a prospective observational study of consecutive patients aged 16 and above referred to cardiovascular MRI (CMR) between January 2012 and June 2013. The MRI scans included evaluation of suspected or manifest diseases such as ischaemic heart disease, non-ischaemic heart disease (mainly hypertrophic/dilated cardiomyopathy, myocarditis, and arrhythmogenic right ventricular cardiomyopathy (ARVC)), aortic diseases, and patients taking part in other cardiovascular MRI studies (study patients). Demographic data was collected and patients categorized into three clinical groups based on the conclusions from the CMR radiologist: Cardiac disease, aortic disease or no findings/study patient. The regional ethical board approved the study (REK 2011/748) and participants gave their written consent. Patients received neither compensation nor benefits from participation in the study.

### MRI scan

We used 1.5 T MRI scanners (Philips Achieva/Philips NT Intera, Philips Healthcare, Best, the Netherlands) for all MRI examinations. Patients were lying supine with the arms along their sides, and held their breath during the scan. To facilitate surface landmark identification, five fish oil softgels were placed along the INL. Fish oil softgels consist mainly of fat and are easily detectable on MRI without causing artifacts. Only the survey images, consisting of 8–10 mm axial scans used for planning the remaining MRI exam, were used in our study. All MRI images were anonymized before export and analysis.

### Measurements

**Fig. 1:**
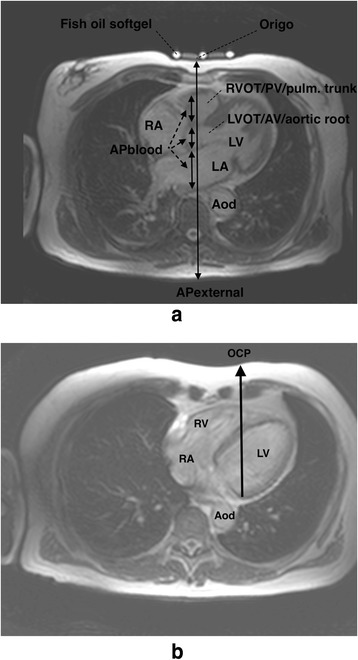
**a** The internipple line (INL) image showing AP_EXTERNAL_ and AP_BLOOD_ (the sum of blood-filled structures along AP_EXTERNAL_.) Fish oil softgels identify the INL level. Abbreviations: Aod, descending aorta; AP_BLOOD_, Anterior-posterior diameter of blood-filled structures; AP_EXTERNAL_, external anterior-posterior diameter; INL, internipple line; LA, left atrium; LV, left ventricle; LVOT/AV/aortic root, left ventricular outflow tract/aortic valve/aortic root; Origo, centre of sternum at the level of the INL; RA, right atrium; RVOT/PV/pulmonary trunk, right ventricular outflow tract/pulmonary valve/pulmonary trunk. **b** Optimal compression point (OCP) image showing how OCP was identified. OCP is the surface projection of a line with maximum sum of left and right ventricle AP diameters. Abbreviations: Aod, descending aorta; LV, left ventricle; OCP, optimal compression point; RA, right atrium; RV, right ventricle

We defined the axial survey image with the fish oil softgels as the INL image, and defined origo as the skin surface above the centre of sternum in this image (Fig. 1a). The external AP diameter (AP_EXTERNAL_) was perpendicular to the skin and represented the distance between origo and the skin dorsally. The left ventricular outflow tract (LVOT), aortic valve (AV), and aortic root are anatomically closely related, and were therefore joined in a common anatomical structure called LVOT/AV/aortic root. The right ventricular outflow tract (RVOT), pulmonary valve (PV), and pulmonary trunk were joined in the anatomical structure RVOT/PV/pulmonary trunk for the same reason. We identified the dominating structure as the structure with the largest AP diameter in both the INL image and the adjacent images caudal and cephalad combined. Further, in each patient, we quantified the inner diameters of the intrathoracic blood-filled structures along the AP_EXTERNAL_ in the INL image (Fig. 1a). The sum of the inner diameters was called the AP diameter of blood-filled structures (AP_BLOOD_). We calculated the ratio between AP_BLOOD_ and AP_EXTERNAL_.

Finally, we defined a hypothesis generating optimal compression point (OCP) as the ventral skin surface projection of a line perpendicular to the skin dorsally (Fig. 1b) in the axial image where LVOT was not present, and where the sum of left and right ventricle AP diameters was as large as possible. We defined this image as the OCP image. The distance from the origo (INL image) to the OCP (OCP image) in the coronal plane was calculated using coordinates provided by Osirix software. All measurements were done by one of the authors (SN) using OsiriX software (v.5.9 32-bit by Pixmeo SARL, Bernex, Switzerland). An experienced CMR radiologist (KHS) performed review of 56 % of the cases, based on the initial registrations and measurements.

### Statistical analysis

All variables are reported as mean (SD) or median (IQR), as appropriate based on normality tests and compared using Student’s *t*-test or Mann–Whitney *U*-test accordingly for continuous data, and Chi-squared tests with continuity correction for categorical data. We performed multiple regression analyses of the dependent variables; AP_EXTERNAL_, AP_BLOOD_, and the AP_BLOOD_/AP_EXTERNAL_-ratio to find possible explanatory factors in gender (male, female), age (continuous), height (continuous), and clinical groups (cardiac disease, aortic disease or no findings/study patient). We used SPSS v 22 (SPSS Inc., Chicago, IL, USA) for all analyses.

## Results

### General characteristics of the patients

During the 18-month study period, 149 consecutive patients underwent 151 MRI scans. None denied participation. Seven cases were excluded due to repeat MRI scans in patients already included (*n* = 2), MRI scans not available (*n* = 2), INL could not be identified (*n* = 2), or unavailable case report form (*n* = 1). Table 1 displays clinical characteristics of the 144 patients enrolled.

**Table 1:** Patient characteristics (*n* = 144)

Age (years)	51.8 ± 17.2
Males	110 (76 %)
Height (cm)	177.5 ± 9.0
Weight (kg)	81.0 ± 14.1
BMI (kg/m^2^)	25.6 ± 3.6
Clinical groups:	
Cardiac disease	74 (51 %)
Aortic disease	13 (9 %)
No findings/study patient	57 (40 %)

Nonpercent values are ± SDs

*BMI* body mass index

### The structures beneath the centre of sternum

**Fig. 2:**
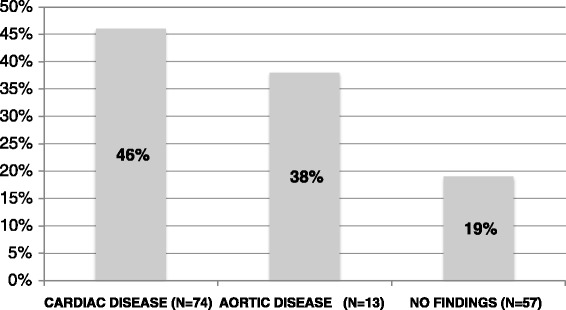
Presence of LVOT/AV/aortic root beneath the centre of sternum at the internipple line level divided into clinical groups. Abbreviations: LVOT/AV/aortic root, left ventricular outflow tract/aortic valve/aortic root

We described the presence of all blood-filled structures beneath the centre of sternum in the INL image. The LVOT/AV/aortic root was present in almost half (46 %) of patients with cardiac disease vs. 19 % of patients with no findings/study patients (*p* = 0.006) (Fig. 2). The left and right ventricle was present in 2 and 48 % of all patients, respectively. Details are described in Additional file 1. The most frequent dominating structure in all patients was the left atrium (41 %) or the right ventricle (31 %). The left ventricle was not the dominating structure in any patient.

### Anterior-posterior diameters

**Fig. 3:**
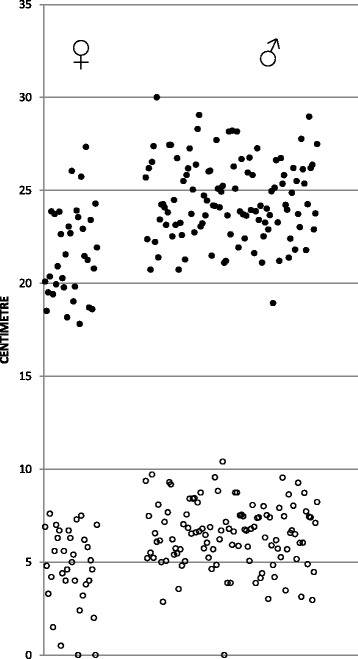
Scatter plot of AP_EXTERNAL_ and AP_BLOOD_ at the internipple line level, divided into gender (left – females, right – males). Each patient is represented by one AP_EXTERNAL_ symbol (filled) and one AP_BLOOD_ symbol (open). APBLOOD, Anterior-posterior diameter of blood-filled structures; APEXTERNAL, external anterior-posterior diameter

Mean (SD) AP_EXTERNAL_ for males and females were 24.5 (2.2) and 21.6 (2.4) cm, respectively. A compression depth of 5–6 cm would correspond to a reduction in AP_EXTERNAL_ for males and females of 20–24 % and 23–28 %, respectively. AP_BLOOD_ for males was 6.5 (1.8) cm and for females 4.7 (2.1) cm with AP_BLOOD_/AP_EXTERNAL_–ratio of .26 and .22 respectively. Unadjusted and adjusted differences between males and females are displayed in Table 2. Gender and age were significant explanatory factors for AP_EXTERNAL_, whereas for AP_BLOOD_ and AP_BLOOD_/AP_EXTERNAL_-ratio only gender was significant in the regression analysis. Figure 3 displays a scatter plot of AP_EXTERNAL_ and AP_BLOOD_ at the internipple line level, divided into gender.

**Table 2:** The unadjusted and adjusted differences between males and females in AP_EXTERNAL_, AP_BLOOD_ and the ratio between them

	Mean difference (95 % CI)	Difference (Adj.)^a^	*P*-value (regression analysis)
AP_EXTERNAL_			
Gender	2.9 (2.0, 3.8)	2.1 (1.1, 3.2)	<0.001
AP_BLOOD_			
Gender	1.8 (1.1, 2.5)	2.0 (1.0, 2.9)	<0.001
AP_BLOOD_/AP_EXTERNAL_			
Gender	0.05 (0.01, 0.08)	0.06 (0.02, 0.10)	0.003

^a^Adjusted for height, age and clinical group

### Hypothetical optimal compression point (OCP)

**Fig. 4:**
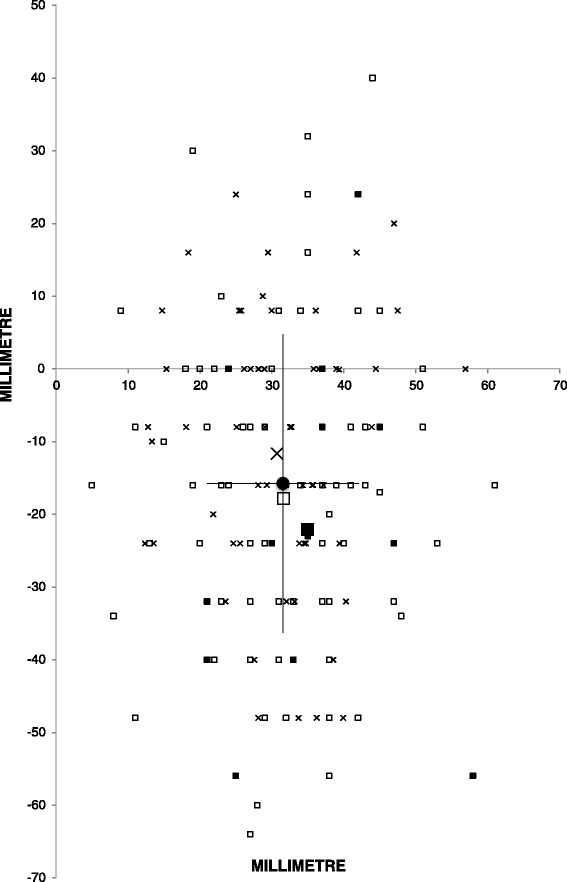
Optimal compression point (OCP) in the coronal plane based on maximum compressible left and right ventricle. Origo is the centre of sternum at the internipple line level. One small symbol represents one patient and clinical groups mean values with large symbols (cardiac disease ⧠ (open square), aortic disease █ (filled square), and no findings/study patient x (crosses)). Overall mean (filled circle) represented with standard deviations

Figure 4 displays OCP for all patients and clinical groups. Mean (SD) OCP was found 3.2 (1.1) cm to the left and 1.6 (2.1) cm caudal to origo. There were no significant differences between clinical groups, but a tendency to larger caudal displacement of OCP in the clinical groups with pathological findings (cardiac disease and aortic disease).

## Discussion

In 144 patients referred to cardiovascular MRI, the relationship between intrathoracic cardiovascular structures and detectable surface landmarks for chest compression hand placement varied significantly. We found that left ventricle was rarely present beneath centre of sternum while the left ventricular outflow tract (LVOT), aortic valve, or aortic root was present in almost half of the patients with cardiac disease.

External chest compressions have been integral to CPR since first described by Kouwenhoven in 1960 [8]. Recommendations for hand position have changed over the years. ILCOR recommends placing the hands on the lower half of the sternum. ERC/ILCOR concluded in 2010 that the internipple line (INL) was not a reliable landmark for hand placement based on diverging results from studies of CT-scans and simulations on real patients. Shin et al. found the ascending aorta, the root of aorta or the LVOT beneath INL in about 80 % of the patients and concluded that compressing the sternum more caudally might be more effective [3]. However, Kusunoki et al. concluded that use of the INL for CPR might result in the compressing hand extending to the xiphoid process with organ injury as a possible consequence [9]. Additionally, the lower half of the sternum might not be readily understandable for a layperson while “in the middle of the chest, between the nipples” is easily understandable. Birkenes et al. found that the use of INL in pre-arrival telephone instructions to rescuers resulted in less caudal hand placements and none in the abdominal region [10].

In the present study the fraction of patients with LVOT/AV/aortic root was lower than found by Shin et al. [3], but still occurred in almost half the patients with cardiac disease, less in the other clinical groups. Patients in both studies were supine with arms along the sides. The LV and RV were present beneath the centre of sternum in 3 and 41 % in patients with cardiac disease, respectively; in contrast to Shin et al. who found LV present in one-fifth in their combined group. Papadimitriou et al. used CT scans to investigate the relationship between cardiac structures and ribs and found that the occurrence of cardiac chambers under the lower part of the sternum (ribs 4–6) was very high [11], almost 100 % for RV and LV. They concluded that this was a reasonable position for hand placement during chest compressions. In contrast to our study they noted all structures that appeared in the CT axial scan. We chose to identify only structures directly beneath the centre of sternum, although other structures were present in the INL image. This can partly explain the diverging results between their study and the present study.

Forward blood flow is provided by direct compression of the cardiac ventricles and intra-thoracic pressure fluctuations. Compressing the LVOT might reduce the positive effect of compressing the ventricles, and asymmetrical compression of the ventricles (RV > LV) is an unknown factor. The combination of these might explain the lack of effect of chest compressions sometimes observed clinically, and the heterogeneity observed in a clinical pilot study of changing hand positions [12]. In that study, where endtidal CO2 (EtCO2) was used as an indication of cardiac output during CPR, there were no significant differences in EtCO2 with altered compression points. Direct observations with transoesophageal echocardiography (TEE) by Hwang et al. during resuscitation showed that the outflow of the left ventricle is affected resulting in varying degrees of narrowing in the LVOT and/or the aortic root [4]. They also suggested that the technical limitations of TEE preclude its use during routine CPR. Further, Tømte et al. used TEE in anaesthetized domestic pigs and revealed the presence of asymmetrical compression in several animals despite the same sternal piston positioning and the same placement of the pigs with prevention of lateral displacement [13].

To our knowledge, few others have hypothesized an optimal compression point based on the cardiovascular structures. We found a hypothesis generating optimal compression point (OCP) based on maximum compressible left and right ventricle to be to the left and caudal to the centre of sternum at the INL level. Shin et al. argued for a more caudal compression point [3], and in children the best compression point has been found to be the lower third of sternum, caudal to the INL [14]. If the present hypothesis is correct, in a majority of patients the optimal compression point is both to the left of the midline and slightly caudal to the INL. As the LVOT anatomically, in a normal setting, is directed rightward and cranially, this is also an argument for changing the compression point leftwards. It is not fully established how the force from chest compressions is transferred to intrathoracic structures. Pickard et al. suggested a mechanism where the sternum acts as a hinge as it is relatively fixed cranially making the caudal part of the sternum more flexible [7]. As to moving the compression point lateral to the sternum, we are concerned that compressions might not squeeze the intrathoracic structures between the sternum and the vertebral column, a concern only relevant if cardiac pump theory is the dominant blood flow mechanism. In the search for optimal hand position in an individualised CPR by professionals, we believe further research is warranted to develop a strategy when standard CPR is not successful. We found some differences between the clinical groups and we speculate that this can be further explored in clinical studies, as pre-existing cardiac disease as a broad category would be known on scene in many patients, and therapy could be changed accordingly to reduce the risk of compressing directly over the LVOT.

The recommended chest compression depth changed from 38–52 mm to 5–6 cm [2] partly based on didactic considerations and partly on extrapolation of earlier studies [15–18], but recent results questions this and has found the optimum chest compression depth to be 40–55 mm for both men and women [3, 19]. Current ERC guidelines recommend a compression depth of 5 cm, not exceeding 6 cm in an average adult [1]. The present study was conducted based on guidelines from 2010. We found that compressing the recommended 5 cm corresponded to 20 and 23 %, of AP_EXTERNAL_ for males and females respectively. This is in agreement with Pickard et al. who showed that a 4–5 cm reduction corresponded to approximately one-fifth of the AP diameter [7]. In children, Braga et al. found that in some age groups there would be no room for intrathoracic structures between the sternum and columna when compressing the recommended one-third of the AP diameter, and were concerned that this might be harmful [20].

If it can be assumed that it is the squeezing of blood-filled structures that cause blood-flow, the present results indicate that 5 to 6 cm compressions are possible and can be valuable in most males. But, as mean AP_BLOOD_ for females was only 4.7 cm at the centre of sternum, other structures than blood might be compressed. An observational study by Hellevuo et al. suggested that a compression depth of more than 6 cm in male patients is associated with an increased rate of injury in adults when compared with compression depths of 5–6 cm during CPR [21]. No such association was observed in female patients. Although there is little direct evidence that damage from chest compression is related to compression depth, there might not be haemodynamic benefits from compressing non-blood-filled structures such as connective tissue and myocardium. Chest compressions and artificial ventilation during CPR might relocate intrathoracic structures. With aortography, Niemann et al. showed in a canine experimental study, that the cardiac silhouette is displaced posteriorly during chest compressions [22]. MRI survey scans were not done during CPR. However, we found only cardiac and mediastinal structures along AP_EXTERNAL_ (Fig. 1a). These effects might not be relevant for blood flow generated by a thoracic pump mechanism. ERC guidelines do not differentiate between gender, body size or medical history. We believe the upper limit for chest compression depths, especially in women, should be elucidated.

Limitations apply to this study. It is a prospective, observational single-centre study and thus is limited by the patient selection. While fewer females than males participated, the proportion matches the ratio for cardiac arrest victims under the age of 65 [23]. The ethnicity of patients was not noted, yet Norwegians are mostly caucasians. Our results may not be representative for other ethnicities. The survey scans used in our study were obtained without electrocardiogram gating. As the axial scans are 8–10 mm thick and sometimes include motion artifacts, this creates some uncertainty to our results. All measurements were done by one author, with additional aid by an experienced radiologist in 56 % of the cases, but no formal interobserver variation was calculated. Structures that laid adjacent to AP_EXTERNAL_, but were not pierced by it (since the line was thin), have not been identified as a structure beneath the centre of sternum. This explains why AP_BLOOD_ in some of the cases was zero. Further, chest compressions by hand or mechanical device transfer pressure to a wider area than the thin line we used for measurements. Hence, volumetric measurements would provide more accurate calculations than our method. Extrathoracic soft tissue might be compressed during CPR, affecting AP_EXTERNAL_. But as our patients were lying supine, the soft tissue posteriorly is already compressed to some extent.

## Conclusions

The left ventricular outflow tract/aortic valve/aortic root was found beneath the centre of sternum at the internipple line (INL) level in almost half the patients with cardiac disease, while the left ventricle was present in only 3 %. Recommended chest compression depths exceeded the anterior-posterior diameter of blood-filled cardiovascular structures in more than half of the females. A hypothesis generating optimal compression point was found 3.2 cm to the left and 1.6 cm caudal to the centre of sternum at the INL level. Based on our study, individualized compression point and depth could be further studied in a prospective, clinical study.

## Additional file

Additional file 1: The presence of blood-filled structures beneath the centre of sternum at the internipple line level. Percentages do not sum up to 100 because several structures may be present in one patient. (DOCX 65 kb)

## Abbreviations

AP: anterior-posterior
AP_BLOOD_: AP diameter of blood-filled structures
AP_EXTERNAL_: external AP diameter
ARVC: arrhythmogenic right ventricular cardiomyopathy
AV: aortic valve
CMR: cardiovascular MRI
CPR: cardiopulmonary resuscitation
INL: internipple line
LV: left ventricle
LVOT: left ventricular outflow tract
OCP: optimal compression point
PV: pulmonary valve
RV: right ventricle
RVOT: right ventricular outflow tract
TEE: transoesophageal echocardiography
