# Supplementary material for: Radiological assessment of chest compression point and achievable compression depth in cardiac patients
Source: Scand J Trauma Resusc Emerg Med. 2016 Apr 22;24:54. doi: 10.1186/s13049-016-0245-0 (PMC4840890; doi:10.1186/s13049-016-0245-0)
Supplement: Additional file 1: — The presence of blood-filled structures beneath the centre of sternum at the internipple line level. Percentages do not sum up to 100 because several structures may be present in one patient. (DOCX 65 kb) [file 13049_2016_245_MOESM1_ESM.docx]

Additional file 1
The presence of blood-filled structures beneath the centre of sternum at the internipple line level. Percentages do not sum up to 100 because several structures may be present in one patient.

|  | All patients (n=144) | Cardiac disease (n=74) | Aortic disease (n=13) | No findings/ study patient (n=57) |
| --- | --- | --- | --- | --- |
|  | n(%) | n(%) | n(%) | n(%) |
| Left atrium | 100 (69) | 55 (74) | 11 (85) | 34 (60) |
| Left ventricle | 3 (2) | 2 (3) | 0 (0) | 1 (2) |
| Right atrium | 55 (38) | 23 (31) | 4 (31) | 28 (49) |
| Right ventricle | 69 (48) | 30 (41) | 3 (23) | 36 (63) |
| LVOT/AV/ aortic root | 50 (35) | 34 (46) | 5 (38) | 11 (19) |
| Ascending aorta | 8 (6) | 4 (5) | 2 (15) | 2 (4) |
| Descending aorta | 11 (8) | 8 (11) | 1 (8) | 2 (4) |
| RVOT/PV/pulm. trunk | 17 (12) | 11 (15) | 2 (15) | 4 (7) |
| Other structures | 5 (3) | 3 (4) | 1 (8) | 1 (2) |

Abbreviations:

LVOT/AV/aortic root = Left ventricular outflow tract/Aortic valve/aortic root. Other structures= Other blood-filled structures; RVOT/PV/pulm. trunk = Right ventricular outflow tract/Pulmonary valve/pulmonary trunk.
